# Decoding the functional landscape of long non-coding RNAs in hepatocellular carcinoma: molecular mechanisms, clinical implications, and therapeutic prospects

**DOI:** 10.1186/s43046-026-00380-9

**Published:** 2026-06-29

**Authors:** Humera Naveed, Zain-Ul Abidien, Kaleem Maqsood, Iram Amin, Muhammad Shahid, Samia Afzal

**Affiliations:** University of the Punjab, Lahore, Pakistan

**Keywords:** Hepatocellular carcinoma, Long non-coding RNA, HULC, HOTAIR, MALAT1, PI3K/AKT/mTOR, ceRNA, Biomarker, Therapeutic target

## Abstract

Hepatocellular carcinoma (HCC) remains the third leading cause of cancer-related mortality worldwide, with limited treatment efficacy and poor prognosis, particularly in advanced-stage disease. Despite progress in diagnostic imaging and systemic therapies, early detection and effective targeted interventions remain major clinical challenges. Long non-coding RNAs (lncRNAs), a class of regulatory RNA molecules exceeding 200 nucleotides in length, have emerged as essential regulators of oncogenic signaling, metabolism, and tumor microenvironment in HCC. This review provides a comprehensive overview of the molecular mechanisms, biological functions, and clinical relevance of key lncRNAs involved in HCC progression. We summarize the dual roles of lncRNAs as oncogenic drivers and tumor suppressors, with particular emphasis on oncogenic lncRNAs including CYTOR, UCA1, MALAT1, SPRY4-IT1, uc001ncr, AF085935, HULC, and HOTAIR. Their regulatory functions are discussed within major cancer-associated signaling networks, including the PI3K/AKT/mTOR, Wnt/β-catenin, TGF-β/NF-κB, and EMT-associated pathways. Furthermore, each lncRNA is discussed in the context of its expression pattern, endogenous RNA (ceRNA) interactions, molecular partners, and functional contributions to cellular proliferation, invasion, metastasis, angiogenesis, immune modulation, and therapeutic resistance. In addition, we explore the emerging potential of lncRNAs as minimally invasive diagnostic and prognostic biomarkers, owing to their stability and detectability in tissue and circulating biofluids. We also discuss recent advances in RNA- targeted therapeutic strategies, including RNA interference, siRNA-mediated silencing, antisense oligonucleotides, and CRISPR-based genome editing approaches. Collectively, this review highlights the importance of integrating lncRNA expression signatures into personalized HCC management and the promising role of lncRNA-based diagnostics and therapeutics in transforming the future landscape of liver cancer detection, prognosis, and treatment.

**Supplementary Information:**

The online version contains supplementary material available at 10.1186/s43046-026-00380-9.

## Overview of Hepatocellular Carcinoma (HCC)

### Global and regional epidemiology of HCC

Hepatocellular carcinoma (HCC) is the most prevalent primary liver cancer and a major global health concern due to its high mortality and poor prognosis [1]. It is strongly associated with chronic liver diseases, including hepatitis B virus (HBV) and hepatitis C virus (HCV) infections, chronic alcohol use, and metabolic disorders such as metabolic dysfunction-associated steatotic liver disease (MASLD) [1, 2]. Globally, HCC shows striking geographical variation, with approximately 80% of cases occurring in East Asia and sub-Saharan Africa [3], where HBV and HCV are endemic—China alone accounts for about 55% of global cases [4]. In 2018, liver cancer had an estimated incidence of 9.3 and mortality of 8.5 per 100,000 person-years [5], reflecting consistently poor survival outcomes [6]. In Western countries, including North America and Europe, HCC incidence has been rising, driven by alcohol use, obesity, MASLD, and type 2 diabetes. Unlike viral or alcohol-related HCC, metabolic-related HCC can develop even in non-cirrhotic livers. Regionally, East Asia and sub-Saharan Africa remain the highest-burden areas, while the lowest rates (< 3 per 100,000) are reported in Australia and parts of Northern Europe [7]. These epidemiological disparities underscore the importance of region-specific biomarker development and lncRNA profiling—areas particularly relevant to high-burden regions such as Pakistan, where HBV-related HCC constitutes a substantial disease burden.

### Etiological factors (HBV, HCV, alcohol, aflatoxin, metabolic syndrome)

Hepatocellular carcinoma arises from multiple risk factors that cause chronic liver injury, inflammation, and genetic alterations. Table 1 summarizes the major etiological factors, including HBV [8], HCV, chronic alcohol use, aflatoxin exposure, and metabolic disorders such as obesity, diabetes, and non-alcoholic fatty liver disease (NAFLD). HBV is a leading global cause, particularly in East Asia and sub-Saharan Africa, promoting HCC through chronic hepatic inflammation and direct integration of viral DNA into the host genome, inducing genomic instability and oncogenic mutations [9]. HCV causes chronic hepatitis and cirrhosis via persistent inflammation and oxidative stress. It does not integrat**e** into host DNA but remains a major cause of HCC in Western and some Asian countries [10]. Aflatoxins (toxic compounds from mold-contaminated food) cause p53 gene mutations and act synergistically with HBV to increase HCC risk, especially in Africa and Asia [11]. Metabolic syndrome, including obesity, diabetes, and NAFLD/NASH, is an emerging HCC driver in developed regions, promoting liver carcinogenesis via insulin resistance, lipid accumulation, and chronic inflammation [12].

**Table 1:** Major etiological factors contributing to hepatocellular carcinoma (HCC) development

Etiological Factor	Mechanism of HCC Development	Regional Importance	References
HBV	Chronic inflammation, Viral DNA integration, and genome instability	High in East Asia, Sub-Saharan Africa	[13]
HCV	Chronic hepatitis, inflammation, fibrosis, cirrhosis	Significant in Western countries, parts of Asia	[14]
Alcohol	Oxidative stress, Liver cirrhosis from chronic damage	Global, prominent in Western countries	[15]
Aflatoxins	DNA mutation (p53 inactivation), synergistic with HBV	Sub-Saharan Africa, Asia	[16]
Metabolic Syndrome	Insulin resistance, inflammation, fibrosis, HCC without cirrhosis	Rising globally, especially in Western countries	[17]

### Molecular pathogenesis of HCC

**Fig. 1:**
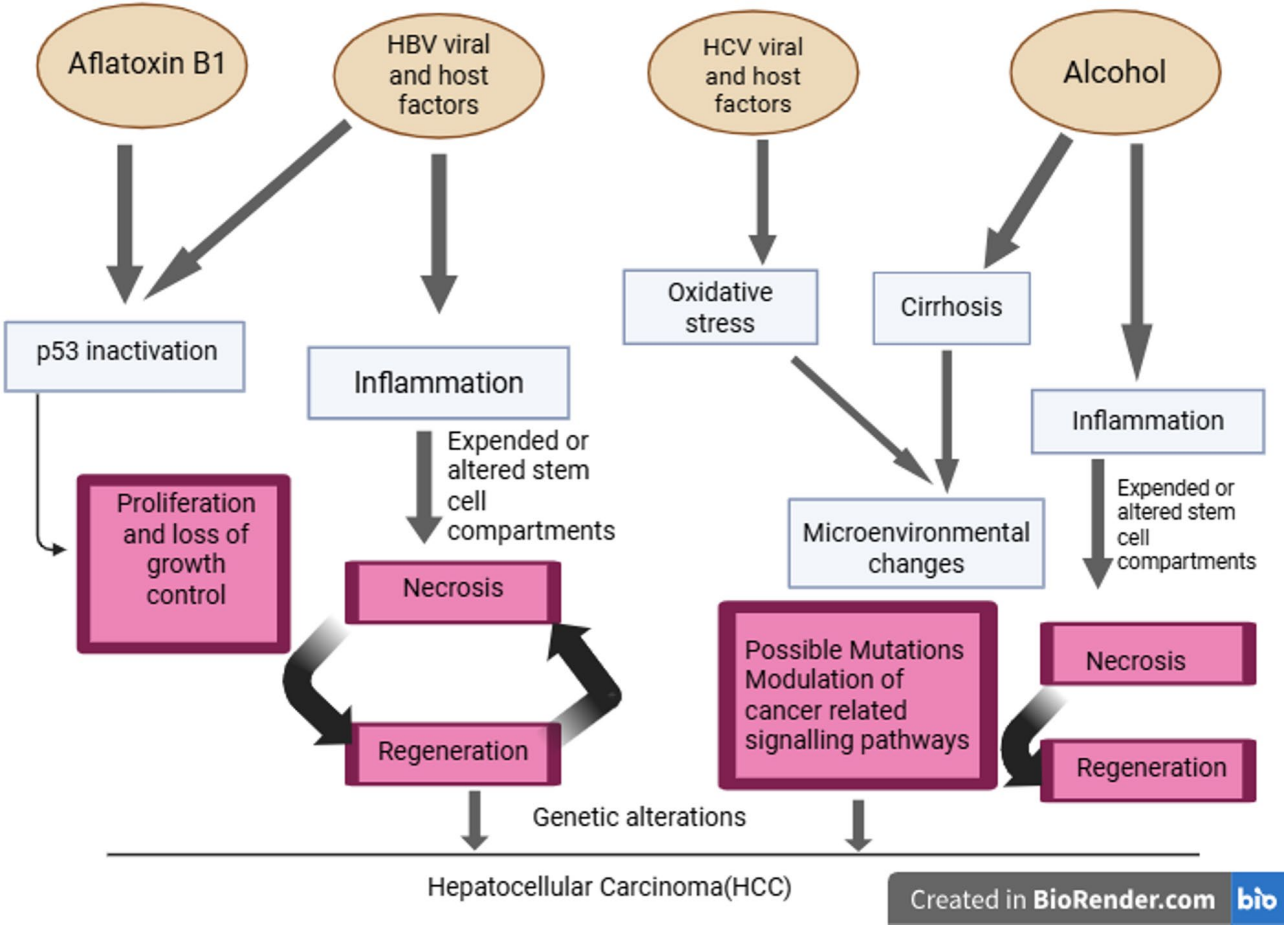
Schematic illustration of the major etiological factors contributing to hepatocellular carcinoma (HCC). Aflatoxin B1 causes p53 inactivation, leading to uncontrolled proliferation, while HBV and HCV infections trigger chronic inflammation, oxidative stress, and microenvironmental changes that promote mutations and signaling pathway alterations. Alcohol consumption further contributes to cirrhosis, inflammation, and necrosis–regeneration cycles, all of which drive genetic alterations and hepatocarcinogenesis

HCC develops through genetic, epigenetic, and microenvironmental alterations, often associated with chronic HBV or HCV infection. Chronic liver injury progressively increases mutational burden. In HBV-related HCC, viral DNA integration into host oncogenes leads to genomic instability and uncontrolled cellular proliferation [18]. Oncogenic signaling pathways dysregulation, epigenetic reprogramming, and the immunosuppressive tumor microenvironment collectively support carcinogenesis, while cancer stem cells contribute to heterogeneity and therapy resistance [18]. The mechanism is illustrated in Fig. 1.

### Current diagnostic and therapeutic limitations

Ultrasound is the standard screening tool for high-risk individuals but has limited sensitivity (~ 60%) for small or early-stage HCC, especially in patients with fatty liver or cirrhosis [19]. Computed Tomography (CT) and MRI provide better sensitivity but are expensive and unsuitable for routine screening due to the risk of radiation exposure and limited accessibility. Contrast-enhanced ultrasound (CEUS) improves lesions characterization but can yield false positive [20]. A biopsy is used when imaging is inconclusive, but it is invasive and subject to sampling error [21]. Alpha-fetoprotein (AFP) remains the most used biomarker but lacks sensitivity for early HCC. Newer candidates, such as circulating tumor DNA (ctDNA) and methylation-based markers, show promise but require extensive clinical validation [22]. Screening efficacy is hampered by overdiagnosis of indolent lesions, poor adherence, and inadequate risk stratification, reducing the detection of clinically significant cases [23]. Most patients are diagnosed at advanced stages, precluding curative interventions such as surgical resection or liver transplantation, which are restricted to early HCC and depend on liver function and donor availability [24]. Even after curative therapy, recurrence rates remain high. Systemic treatments, including sorafenib, lenvatinib, and immune checkpoint inhibitor combinations, benefit only a subset of patients and are associated with resistance, toxicity, and high cost [25]. These limitations collectively underscore the urgent need for novel, validated biomarkers and therapeutic targets, a need that lncRNA research is beginning to address.

## Long non-coding RNAs (lncRNAs): biology, classification and rationale

### Discovery and characteristics of lncRNAs

**Fig. 2:**
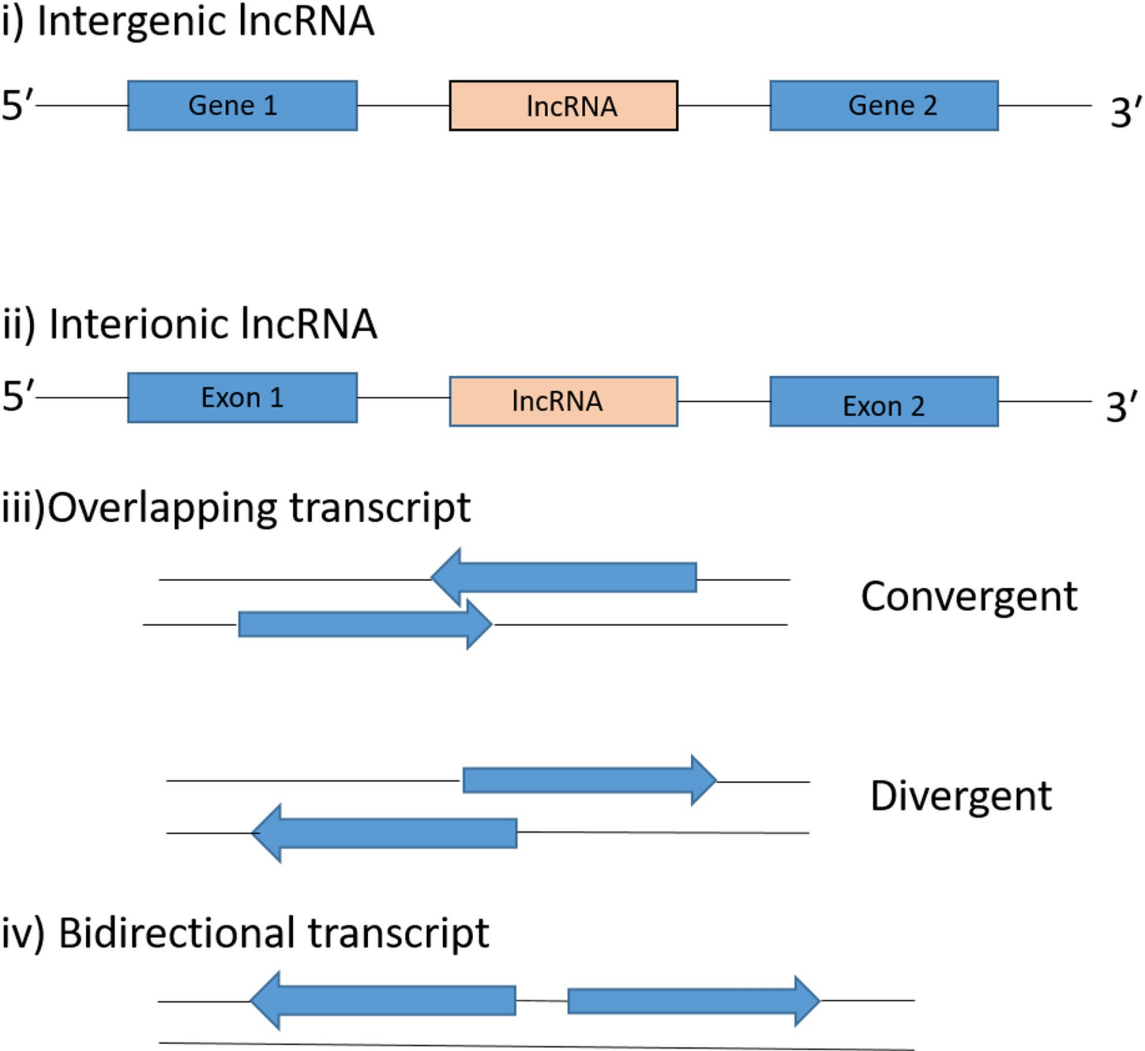
Schematic representation of the major genomic classifications of long non-coding RNAs (lncRNAs). (i) Intergenic lncRNAs are located between two protein-coding genes. (ii) Intronic lncRNAs originate from Intronic regions within a gene. (iii) Overlapping transcripts share partial sequence overlap with nearby genes and may be oriented in a convergent or divergent direction. (iv) Bidirectional transcripts are transcribed from opposite strands in close proximity, usually near a shared promoter region

Long non-coding RNAs (lncRNAs) are RNA transcripts over 200 nucleotides that regulate gene expression without coding for proteins. Initially identified through gene-specific cloning strategies, their systematic discovery expanded substantially with high-throughput sequencing and bioinformatics, revealing thousands of lncRNAs expressed in tissue and context-specific manner across species [26]. Key features of lncRNAs include their length, absence of open reading frames, transcription from distinct genomic loci, epigenetic signatures, functional versatility, and a wide range of biological processes, including metabolism, immunity, neural development, and malignant transformation [27]. A schematic representation of the major genomic classifications of long non-coding RNAs (lncRNAs) is given in Fig. 2.

### Mechanisms of action

LncRNAs regulate gene expression at epigenetic, transcriptional, and post-transcriptional levels through diverse molecular mechanisms. At the chromatin level, lncRNAs recruit histone-modifying complexes such as Polycomb Repressive Complex 2 (PRC2) and LSD1 to specific genomic loci, facilitating H3K27 trimethylation and gene silencing. They can also form RNA-DNA triplex structures at promoters to alter chromatin accessibility [28]. At the transcriptional level, LncRNAs serve as cofactors for transcription factors, modulating their activity or stability. Post-transcriptionally, lncRNAs regulate mRNA splicing, nuclear transport, stability, and translation through binding with mRNAs or RNA-binding proteins [29]. A critical and extensively studied post-transcriptional mechanism is the competing endogenous RNA (ceRNA) hypothesis, whereby lncRNAs act as miRNA sponges to relieve suppression of shared target mRNAs. However, the physiological validity of ceRNA interactions depends on the stoichiometric abundance of the lncRNA, miRNA, and target mRNA—a parameter rarely measured under endogenous conditions and an essential limitation when interpreting in vitro overexpression studies [30].

### Role of lncRNAs in cancer biology

**Fig. 3:**
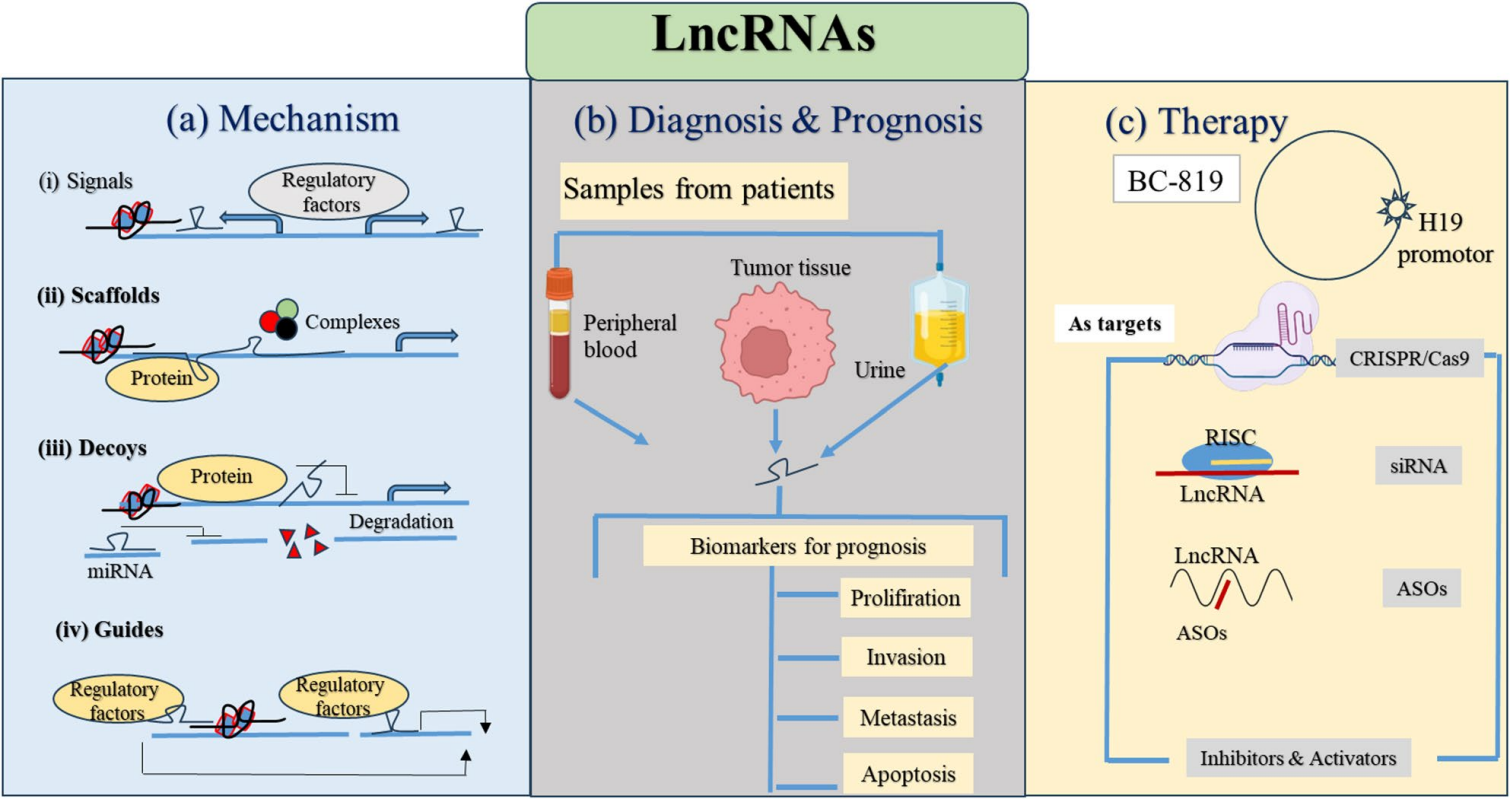
Overview of the diverse roles of long non-coding RNAs (lncRNAs) in cancer biology. **a**Mechanisms: lncRNAs function as signals, scaffolds, decoys, and guides, influencing gene regulation and protein interactions. **b **Diagnosis and prognosis: lncRNAs serve as biomarkers detectable in tumor tissue, blood, or urine, aiding in the evaluation of proliferation, invasion, metastasis, and apoptosis. **c** Therapeutic applications: lncRNAs can act as therapeutic targets or tools, using strategies such as CRISPR/Cas9, siRNA, antisense oligonucleotides (ASOs), or lncRNA-based therapies like BC-819 (H19 promoter) to modulate gene expression and improve treatment outcomes

LncRNAs are central regulators of cancer progression, functioning as oncogenes or tumor suppressors depending on cellular context [31]. Dysregulated lncRNA expression correlates with tumor stage, metastasis, and prognosis across multiple cancer types [32]. LncRNAs influence major oncogenic pathways, contributing to tumor growth and therapy resistance, and actively remodel tumor microenvironment and angiogenesis networks [33]. In the liver, LncRNAs maintain metabolic homeostasis in hepatocytes by controlling lipid metabolism, glucose balance, and energy regulation through pathways including mTOR, AMPK, SIRT6, PPARα, and FOXO1 [34]. Dysregulated lncRNAs contribute to MASLD, fibrosis, and hepatic inflammation modulating apoptosis and inflammatory responses in hepatic stellate cells and Kupffer cells. They promote fibrogenesis and proliferation in hepatic stellate cells, modulate apoptosis, and regulate inflammatory responses in Kupffer cells [35]. Diverse regulatory functions of lncRNAs make them central players in HCC development, metastasis, and treatment response, as illustrated in Fig. 3.

### Rationale for lncRNA selection

The lncRNAs discussed in this review (CYTOR, UCA1, MALAT1, SPRY4-IT1, uc001ncr, AF085935, HULC, and HOTAIR) were chosen to represent the molecular mechanism and clinical features of lncRNA-mediated HCC. Rather than compiling all reported HCC-associated lncRNAs, we focused on molecules that are collectively involved in the major hallmarks of HCC progression, including cell proliferation, invasion, metastasis, angiogenesis, epithelial-mesenchymal transition, and therapeutic resistance. The other reason for selection is that they regulate distinct yet interconnected oncogenic pathways, particularly PI3K/AKT/mTOR [36], Wnt/β-catenin, TGF-β/NF-κB, and EMT-associated signaling [37], thereby enabling a comparative evaluation of convergent regulatory mechanisms. In addition, the selected panel includes both extensively validated lncRNAs with established diagnostic and prognostic significance (HULC, HOTAIR, and MALAT1) and emerging candidates (CYTOR, SPRY4-IT1, uc001ncr, and AF085935) [38, 39] that provide insights into newly identified regulatory networks. Therefore, the selection strategy was based on mechanistic diversity, pathway representation, translational relevance, and the availability of experimental evidence, enabling a focused and integrative analysis of lncRNA-driven regulatory circuits in HCC rather than an exhaustive descriptive catalogue of all reported lncRNAs.

### Literature search strategy

A systematic literature search was performed in PubMed, Scopus, and Web of Science. The search terms were ' long non-coding RNA’, ‘lncRNA’, ‘hepatocellular carcinoma’, and ‘HCC’, separately combined with each lncRNA name (CYTOR, UCA1, MALAT1, SPRY4-IT1, uc001ncr, AF085935, HULC, HOTAIR) and with terms including ‘biomarker’, ‘ceRNA’, ‘therapeutic’, ‘single-cell’, ‘exosomal’, ‘signaling pathway’, and ‘epigenetic’. Studies published up to January 2026 were included. Priority was given to original research articles, meta-analyses, and systematic reviews published in the last 5 years. Studies lacking HCC-specific experimental validation or including fewer than 30 patient samples were critically appraised but included when they provided unique mechanistic insights. Bioinformatics data were retrieved from DIANA-LncBase version 3 (https://diana.e-ce.uth.gr/lncbasev3/; accessed January 2026) and TargetScan version 8.0 (https://www.targetscan.org; accessed January 2026), supplemented by TCGA (GDC Data Release 39.0) and GEO for expression validation data.

## Oncogenic and tumor suppressive lncRNAs in HCC: mechanistic framework

### Systematic framework for oncogenic versus tumor-suppressive lncRNAs

LncRNAs exert fundamentally opposing roles in HCC depending on their regulatory targets and cellular context. Oncogenic lncRNAs activate pathways governing tumor proliferation and stemness- including the Wnt/β-catenin and AKT signaling cascade [40]. Oncogenic lncRNAs facilitate DNA repair, promote malignant progression, and sequester tumor suppressor proteins or microRNAs. On the other hand, tumor-suppressive lncRNAs counteract oncogenic signaling and inhibit HCC growth, invasion, and metastasis [41]. Table 2 provides a structured classification of the reviewed lncRNAs by their functional role, expression pattern, and major pathway involvement.

**Table 2:** Summary of functional roles, molecular mechanisms, classification, and clinical significance of key lncRNAs in HCC

lncRNA	Role	Expression	Major Molecular Mechanisms / Pathways	Clinical Relevance	References
CYTOR (LINC00152)	Oncogenic	↑ Up	NF-κB, EMT; EZH2 interaction; cytoskeletal regulation	Advanced stage, vascular invasion, poor OS; candidate diagnostic marker	[42]
UCA1	Oncogenic	↑ Up	FGFR1–ERK, PI3K/AKT/mTOR; ceRNA (miR-216b); autophagy regulation	Poor prognosis; serum-detectable; therapeutic target candidate	[43]
MALAT1	Oncogenic*	↑ Up	RNA splicing; HIF-1α stabilization; EMT; ceRNA (miR-122, miR-146a)	AUC 0.79 (serum); strong prognostic marker; nanoparticle therapeutic target	[44]
SPRY4-IT1	Oncogenic	↑ Up	EZH2 scaffold; Twist1/Vimentin upregulation; ceRNA (miR-101); TNF pathway	Poor prognosis; serum-detectable; anti-metastatic target	[45]
uc001ncr (TUC338)	Oncogenic	↑ Up	Sp1/α-SMA axis; angiogenesis and fibrosis; TME remodeling	Viral-associated HCC; metastasis predictor; limited clinical validation	[46]
AF085935 (LINC01152)	Oncogenic	↑ Up	Putative ceRNA; viral-context transcriptional dysregulation	Serum-detectable; viral-specific biomarker; limited mechanistic data	[47]
HULC	Oncogenic	↑ Up	miR9/PPARA/ACSL1 lipid axis; CREB/FOXM1; autophagy/ferroptosis	Liver-specific; plasma biomarker; metabolic-therapeutic target	[48]
HOTAIR	Oncogenic	↑ Up	PRC2/LSD1 scaffold; miR-122 silencing; c-Met–Caveolin-1; EMT; stemness	AUC 0.998 (panel + AFP); post-transplant recurrence predictor; LNP target	[49]

* Oncogenic role predominates in most studies; limited evidence from knockout models suggests potential tumour suppressor activity in certain contexts. ↑, upregulated; *OS *overall survival, *TME *tumour microenvironment, *LNP *lipid nanoparticle

**Fig. 4:**
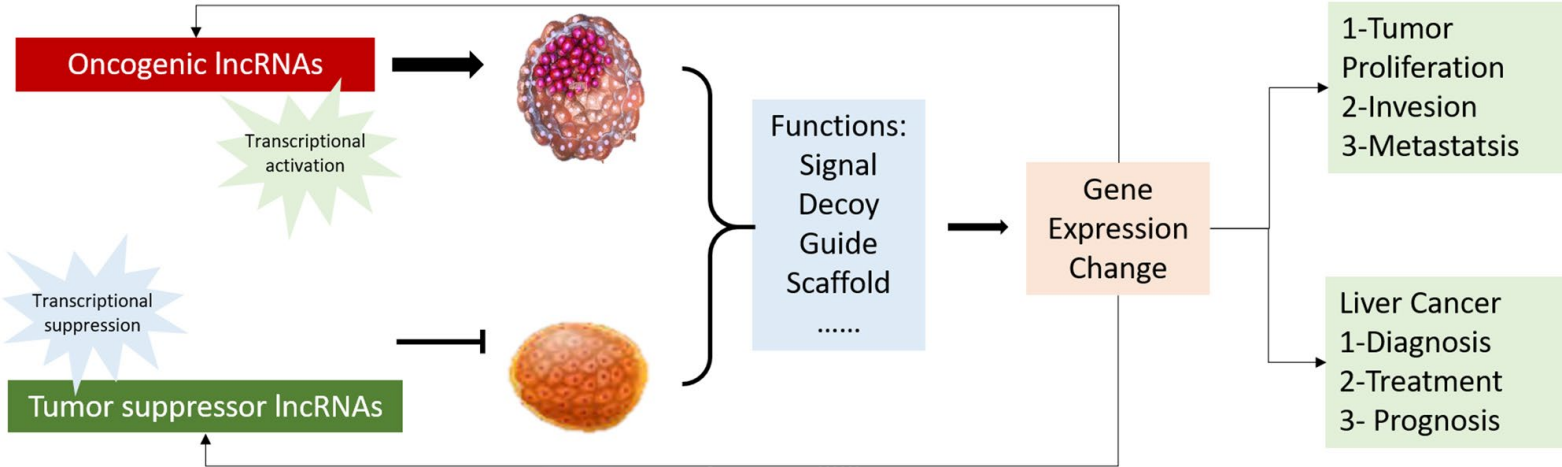
Illustration of the functional roles of oncogenic and tumor-suppressor long non-coding RNAs (lncRNAs) in liver cancer. Oncogenic lncRNAs promote tumor progression through transcriptional activation, while tumor-suppressor lncRNAs are downregulated via transcriptional suppression. Both types can act as signals, decoys, guides, or scaffolds, thereby altering gene expression and influencing tumor proliferation, invasion, and metastasis. These molecular alterations have significant implications for the diagnosis, treatment, and prognosis of liver cancer

A critically underappreciated feature of lncRNA biology in HCC is the regulatory balance that occurs when oncogenic and tumor-suppressive lncRNAs converge on the same signaling pathway. Within the Wnt/β-catenin pathway, oncogenic lncRNAs such as UFC1 and DANCR stabilize β-catenin to promote tumor stemness and self-renewal, while tumor-suppressive counterparts attenuate this activation by sponging β-catenin-activating microRNAs or recruiting repressive chromatin complexes to Wnt target gene promoters [50]. This yin–yang dynamic is equally evident in the PI3K/AKT/mTOR axis, where oncogenic lncRNAs, including UCA1 and ANRIL, enhance AKT/mTOR phosphorylation [51], while tumor-suppressive lncRNAs such as MEG3 and GAS5 attenuate this signaling to restore apoptosis and cell cycle arrest [52]. Similarly, in the NF-κB pathway, CYTOR promotes inflammatory oncogenic signaling while certain suppressive lncRNAs dampen this cascade [53]. This regulatory balance illustrates that lncRNA dysregulation in HCC does not simply alter expression levels of individual molecules but fundamentally shifts the equilibrium of competing regulatory networks, highlighting co-targeting strategies as a rational therapeutic direction. Figure 4 provides an integrated illustration of these functional roles.

### Mechanistic pathways linked to lncRNAs

The PI3K/AKT/mTOR pathway is one of the most critical oncogenic axes in HCC, controlling cell proliferation, survival, migration, invasion, autophagy, and drug resistance [54]. Several lncRNAs modulate this pathway by influencing its core components, such as ANRIL, activating mTOR signaling, UCA1, enhancing AKT/mTOR phosphorylation, and HAGLROS sponges miR-100-5p, increasing mTOR expression [55]. The Wnt/β-catenin signaling pathway, essential for cancer stem cell maintenance and tumor progression, is regulated by lncRNAs including UFC1 and DANCR through stabilization of β-catenin or promotion of its methylation [50].

The NF-κB pathway, a master regulator of inflammation-driven cancer progression and immune evasion, is modulated by lncRNAs including CYTOR and HOTAIR [56]. In HCC specifically, NF-κB activation drives transcription of pro-tumorigenic cytokines including IL-6, TNF-α, and CXCL8, promotes Bcl-2-mediated apoptosis resistance, and stimulates PD-L1 expression to suppress anti-tumour immune responses. CYTOR activates this cascade through EZH2-mediated chromatin remodeling, while HOTAIR sustains it by modulating NF-κB target gene methylation status. They also regulate epithelial-mesenchymal transition (EMT) and metastasis through interactions with TGF-β, Wnt/β-catenin, and STAT3 pathways, and influence TGF-β/Smad signaling critical for tumor progression [57]. The ceRNA network—whereby lncRNAs sponge miRNAs to relieve repression of shared target mRNAs—represents a dominant mechanistic theme among the reviewed lncRNAs [58].

**Fig. 5:**
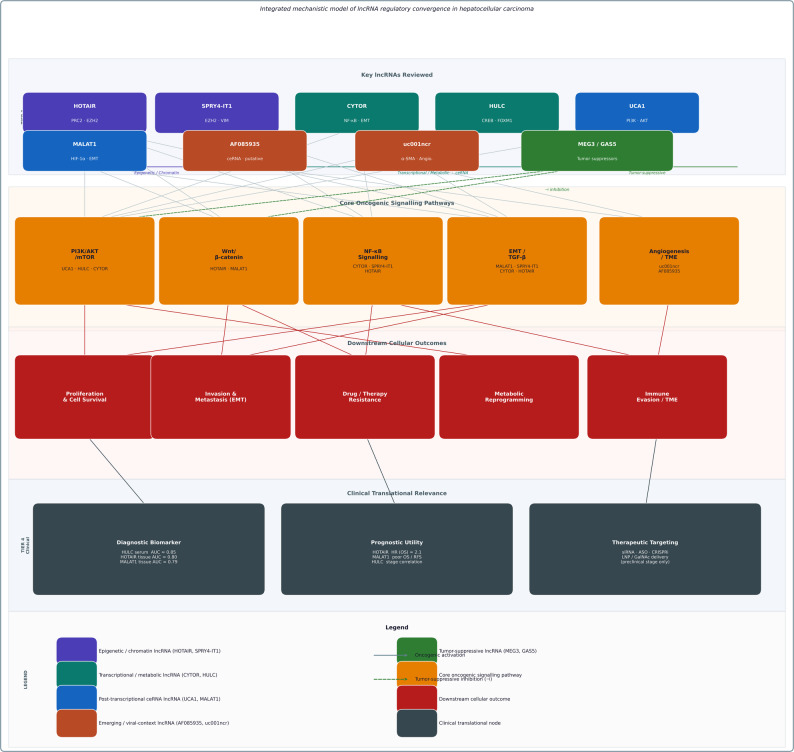
Integrated mechanistic model of lncRNA regulatory convergence in hepatocellular carcinoma. The eight reviewed lncRNAs converge on five core oncogenic signaling pathways driving downstream cellular outcomes of proliferation, invasion, drug resistance, metabolic reprogramming, and immune evasion. Green tumor-suppressive lncRNAs (MEG3, GAS5) exert inhibitory effects on the same pathways. Clinical translational relevance is summarized in the lower tier with reported AUC and HR values. Solid arrows indicate oncogenic activation; dashed arrows indicate tumor-suppressive inhibition. TME, tumor microenvironment; EMT, epithelial–mesenchymal transition; LNP, lipid nanoparticle

However, the physiological relevance of ceRNA interactions critically depends on the stoichiometric abundance of the lncRNA relative to the miRNA pool. Most published ceRNA studies rely on overexpression systems that may not reflect endogenous lncRNA concentrations, introducing a significant confound. Future validation studies should employ CRISPR-based knocking of quantitatively matched expression systems or single-molecule FISH to confirm ceRNA activity at physiological levels. At the epigenetic level, lncRNAs such as HOTAIR and SPRY4-IT1 function as scaffolds guiding the PRC2 complex to specific genomic loci, coordinating H3K27 trimethylation and transcriptional silencing of tumor suppressor genes. The specificity of lncRNA-guided PRC2 targeting—how a given lncRNA directs the complex to one promoter rather than another—remains mechanistically unresolved and represents a key frontier for the field [59]. An integrated mechanistic model connecting lncRNA activity to HCC pathogenesis is depicted in Fig. 5.

## Detailed review of candidate lncRNAs

The eight lncRNAs reviewed below span three distinct regulatory levels — epigenetic scaffolding (HOTAIR, SPRY4-IT1), transcriptional and metabolic control (CYTOR, HULC), and post-transcriptional ceRNA activity (UCA1, MALAT1) — alongside two viral-context candidates with limited mechanistic characterization (AF085935, uc001ncr).

### CYTOR (Cytoskeleton Regulator RNA)

Cytoskeleton Regulator RNA (CYTOR, also known as LINC00152) is an intergenic lncRNA of approximately 852 base pairs with a well-characterized role in cytoskeletal regulation. It exhibits multiple splice isoforms and distinct subcellular localizations that can shift under stress conditions [60]. CYTOR is widely upregulated in various cancers, including hepatocellular carcinoma (HCC), colorectal, lung, pancreatic, and cervical cancers. In HCC specifically, overexpression correlates with advanced tumor stage, lymph node metastasis, vascular invasion, and poor prognosis, making it a valuable diagnostic and prognostic biomarker [61]. CYTOR regulates multiple oncogenic signaling pathways, such as epithelial-mesenchymal transition (EMT) and NF-κB, contributing to tumor growth and metastasis. It also interacts with epigenetic modifiers, such as EZH2, leading to transcriptional reprogramming that enhances proliferation, invasion, glycolysis, inflammatory signaling, immune evasion, and therapeutic resistance [42]. Elevated CYTOR levels are associated with poor survival and aggressive tumor behavior. However, its functional characterization and therapeutic application remain challenging due to the lack of strong sequence conservation across species and multiple transcript isoforms [62].

### UCA-1 (Urothelial Cancer Associated 1)

UCA1 is a non-coding RNA that was initially found in bladder cancer but has since been found to be upregulated in various malignancies, including hepatocellular carcinoma (HCC) [63]. It interacts with RNA-binding proteins and microRNAs and has pyrimidine-rich regions that stabilize the RNA. In HCC, UCA1 acts as a competing endogenous RNA by sponging miR-216b, preventing repression of FGFR1, activating the FGFR1–ERK signaling axis, and facilitating tumor cell survival, growth, and drug resistance [64]. It also regulates autophagy to support cancer cell adaptation under stress conditions. UCA1 overexpression is associated with advanced tumor stage, metastasis, and poor prognosis in HCC and other cancers. Its presence in body fluids suggests its potential as a non-invasive biomarker for early cancer detection and disease monitoring. Targeting UCA1 or its downstream signaling pathways could help suppress tumor progression, overcome therapy resistance, and improve clinical outcomes [65].

### MALAT1 (Metastasis-Associated Lung Adenocarcinoma Transcript 1)

MALAT1 is a conserved and well-characterized long non-coding RNA (lncRNA) involved in RNA splicing, transcriptional regulation, and cancer progression, including hepatocarcinogenesis. It is approximately 8 kb in length and regulates alternative splicing by interacting with serine/arginine-rich splicing factors. In hepatocellular carcinoma (HCC) [66], MALAT1 is significantly upregulated, correlating with tumor proliferation, angiogenesis, invasion, epithelial-mesenchymal transition (EMT), and resistance to apoptosis [67]. MALAT1 functions as a competing endogenous RNA (ceRNA) by sponging tumor-suppressive microRNAs, leading to derepression of oncogenic targets. It stabilizes HIF-1α, amplifying angiogenesis and hypoxic signaling, further promoting tumor aggressiveness [44]. Elevated MALAT1 expression is strongly associated with advanced tumor grade, aggressive phenotypes, and reduced overall survival in HCC patients. It serves as a prognostic biomarker for disease progression and poor outcomes.

### SPRY4-IT1 (Sprouty4 Intronic Transcript 1)

SPRY4-IT1, a long non-coding RNA, plays a significant role in HCC and other malignancies. It is upregulated in HCC, promoting tumor cell proliferation, invasion, and metastasis [45]. It interacts with the epigenetic regulator EZH2, repressing tumor suppressor genes like E-cadherin, facilitating oncogenic transformation. Experimental knockdown suggest that SPRY4-IT1 suppresses HCC cell growth and invasiveness [68]. SPRY4-IT1 also functions as a competing endogenous RNA (ceRNA), sponging miR-101 to relieve repression of target mRNAs involved in oncogenic signaling [69]. Elevated SPRY4-IT1 expression is correlated with poor prognosis and therapeutic resistance in HCC patients. It activates TNF signaling pathways and interacts with the RNA-binding protein HNRNPL, further modulating signaling cascades and EMT progression [70]. Overall, SPRY4-IT1 functions as a potent oncogenic driver in HCC, influencing multiple molecular pathways associated with tumor growth, metastasis, and therapy resistance.

### uc001ncr

Uc001ncr, a long non-coding RNA, has been identified as a potential candidate in liver cancer progression and metastasis [71]. It is significantly upregulated in advanced-stage hepatocellular carcinoma (HCC) and is particularly elevated in virus-associated HCC, particularly in HBV-related cases. Uc001ncr contributes to angiogenesis and tumor microenvironment remodeling by indirectly promoting α-smooth muscle actin (α-SMA) expression. This activation promotes fibrosis, angiogenesis, and extracellular matrix remodeling, facilitating tumor growth and metastatic spread [46]. Experimental knockdown suggests that this lncRNA suppresses cell proliferation, migration, and invasion in vitro and reduces liver metastasis in vivo. These findings suggest that Uc001ncr could be a potential prognostic biomarker and therapeutic target for managing advanced and viral-associated HCC [71].

### AF085935

AF085935, a non-coding RNA, is significantly upregulated in hepatocellular carcinoma (HCC) tissues and patient serum compared to healthy controls. It plays an oncogenic role in liver cancer progression, regulating cancer-related gene expression and indirectly influencing transcriptional and post-transcriptional regulation [72]. It is also elevated in HBV- and HCV-positive HCC patients, with levels correlating with viral infection status and tumor burden. AF085935’s unique overexpression in viral-associated HCC demonstrates its potential as a non-invasive diagnostic and prognostic biomarker, suggesting that it may be a promising candidate for early screening, disease monitoring, and prognosis assessment in HCC patients pending large-scale multicentre validation studies [73].

### HULC (Highly Upregulated in Liver Cancer)

HULC is a liver-specific long non-coding RNA (lncRNA) found in hepatocellular carcinoma (HCC) cells, with its expression predominantly restricted to the liver. It functions in lipid metabolism through the miR-9/PPARA/ACSL1 signaling axis, suppressing miR-9 and preventing PPARA from repressing ACSL1( which stimulates fatty acid metabolism and lipid accumulation in hepatoma cells) [74]. HULC also modulates oncogenic pathways through RNA-protein and RNA-RNA interactions, binding to CREB to enhance its transcription and acting as a competing endogenous RNA by sponging miR-372 and miR-134-5p. Beyond metabolic regulation, HULC influences autophagy and ferroptosis, key processes governing tumor cell survival and therapeutic resistance [75]. HULC’s liver-specific expression, detectability in body fluids, and critical regulatory functions make it a powerful biomarker for diagnosis and prognosis. Targeting HULC or its downstream effectors could be a promising therapeutic opportunities for managing hepatocellular carcinoma by disrupting key metabolic and oncogenic networks.

### HOTAIR (HOX Transcript Antisense RNA)

HOTAIR is a long non-coding RNA that plays a crucial role in epigenetic regulation and hepatocellular carcinoma (HCC) progression. It recruits the Polycomb Repressive Complex 2 (PRC2) and DNA methyltransferases (DNMTs) to specific genomic loci, leading to histone modification and DNA methylation, resulting in the epigenetic silencing of tumor suppressor genes like miR-122 [76]. This silencing forms the molecular basis of HOTAIR-driven hepatocarcinogenesis. HOTAIR regulates multiple oncogenic pathways in HCC, such as the HOTAIR–miR-122–Cyclin G1 and HOTAIR–c-Met–Caveolin-1 signaling axes. It also promotes epithelial-mesenchymal transition (EMT), modulates NF-κB signaling [77], and contributes to chemoresistance by sponging tumor-suppressive microRNAs and altering drug transporter gene expression. Elevated HOTAIR expression is associated with poor prognosis, higher recurrence rates, and shorter survival in HCC patients, particularly those undergoing liver transplantation [78]. Targeting HOTAIR or its downstream signaling pathways could improve treatment response, reduce recurrence, and enhance post-transplant survival in hepatocellular carcinoma. Table 2 summarizes the functional roles, molecular mechanisms, and clinical significance of all the above-discussed lncRNAs.

### Comparative analysis of the eight lncRNAs

The eight reviewed lncRNAs share several mechanistic themes but present important distinctions in clinical validation depth, tissue specificity, and translational readiness. Several independent cohorts of patients with reproducible associations to clinical outcomes, including overall survival, recurrence, and response to therapy, have characterized well-validated molecules such as HULC, HOTAIR, and MALAT1 [79]. Emerging candidates – uc001ncr and AF085935 – are at earlier stages of characterization, supported largely by limited-sized cohort studies (*n* < 100) with incomplete mechanistic dissection [80]. A common mechanistic theme is the convergence of multiple lncRNAs on the PI3K/AKT/mTOR and EMT pathways (UCA1, MALAT1, SPRY4-IT1, HOTAIR, CYTOR), suggesting that these axes are nodes of lncRNA-mediated oncogenic amplification in HCC. The theme of epigenetic regulation (HOTAIR, SPRY4-IT1) sets these two molecules apart as scaffold lncRNAs that physically guide chromatin-modifying complexes, a fundamentally different mechanism from the ceRNA-based activity of HULC, UCA1, and MALAT1 [59]. Unlike most broadly expressed carcinogenic lncRNAs, AF085935 and uc001ncr were first discovered in HBV/HCV-associated HCC cohorts and have received less attention in non-viral HCC contexts. Although additional validation across other etiological subtypes of HCC is necessary, this discovery raises the possibility of a connection with virus-related hepatocarcinogenesis [81]. Table 3 summarizes the genomic information of all the above-discussed lncRNAs.

**Table 3:** Genomic information of selected long non-coding RNAs (lncRNAs)

lncRNA	LncBank ID / Accession	Chr	Best Transcript	Gene Symbol	Genomic Length (bp)	Spliced Length (bp)
HULC	ENST00000560605 / NR_004855	chr6: 86,938,203–86,943,058	NR_004855.2	HULC	4,855	500
HOTAIR	ENST00000592070 / NR_003716	chr12: 54,350,214–54,352,329	NR_003716.3	HOTAIR	2,115	2,158
CYTOR	ENST00000428771 / NR_038377	chr2: 218,893,291–218,894,870	NR_038377.1	CYTOR	1,579	840
UCA1	ENST00000431899 / NR_015379	chr19: 15,909,985–15,914,563	NR_015379.3	UCA1	4,578	1,442
MALAT1	ENST00000534336 / NR_002819	chr11: 65,265,217–65,270,225	NR_002819.2	MALAT1	5,008	8,727
SPRY4-IT1	ENST00000448771 / NR_046473	chr5: 140,747,207–140,749,628	NR_046473.1	SPRY4-IT1	2,421	708
UC001ncr (TUC338)	uc001ncr.1 / TUC338	chr12: 53,758,065–53,758,594	uc001ncr.1	TUC338	529	452
AF085935 (LINC01152)	NR_036499 / LINC01152	chr17: 80,203,140–80,207,215	NR_036499.1	LINC01152	4,075	2,742

*Chr *chromosome, *bp *base pair

## Bioinformatics, multi-omics approaches, and emerging technologies

### ceRNA network analysis and database resources

One of the best methods for detecting changes in gene expression of hepatocellular carcinoma is through bioinformatics approaches [82]. Several online databases are available to study lncRNAs. TCGA (https://www.cancer.gov/ccg/research/genome-sequencing/tcga*)* and GEO (https://www.ncbi.nlm.nih.gov/geo/*)* provide large-scale gene expression data, while StarBase and LncBase help identify interactions between lncRNAs, miRNAs, and mRNAs. These tools are useful for exploring the biological roles and molecular networks of lncRNAs. To investigate the regulatory networks generated by these lncRNAs, we performed ceRNA (lncRNA-miRNA-mRNA) network analysis. The predicted miRNAs for these lncRNAs were chosen from the DIANA-LncBase v3 database (https://diana.e-ce.uth.gr/lncbasev3/, assessed January, 2026*)*. The potential targets of these miRNAs were identified using TargetScan v8.0 (http://www.targetscan.org/assessed January, 2026). A predicted ceRNA network was successfully constructed for six lncRNAs (CYTOR, UCA1, MALAT1, SPRY4-IT1, HULC, and HOTAIR). However, reliable miRNA interaction data for uc001ncr and AF085935 were not available in the queried databases, precluding their inclusion in the computational ceRNA network. This lack of annotated interactions highlights the limited characterization of these lncRNAs and underscores the need for further experimental and bioinformatics studies to elucidate their regulatory mechanisms in hepatocellular carcinoma. The lncRNA expression levels can be quantified experimentally using qRT-PCR or RNA in situ hybridisation; ceRNA interactions can be validated by luciferase reporter assays; and protein–lncRNA interactions can be assessed by RNA immunoprecipitation (RIP) and chromatin immunoprecipitation (ChIP). Table 4 presents the predicted ceRNA axis of the six reviewed lncRNAs. Validated miRNA interaction data for uc001ncr and AF085935 are not yet available in current databases; their inclusion in the main Table 4 is therefore omitted pending experimental confirmation. Supplementary Figures S1–S6 predicted lncRNA–miRNA–mRNA ceRNA interaction networks for CYTOR (S1), UCA1 (S2), MALAT1 (S3), SPRY4-IT1 (S4), HULC (S5), and HOTAIR (S6) in HCC. The predicted interactions suggest that the selected lncRNAs may participate in a complex post-transcriptional regulatory network by interacting with multiple miRNAs and their downstream mRNA targets. However, these interactions are bioinformatical predicted and require further experimental validation to confirm their functional significance in HCC.

**Table 4:** Predicted lncRNA–miRNA–mRNA axes in HCC (selected interactions; DIANA-LncBase v3 and TargetScan v8.0)

lncRNAs	miRNAs involved	Target mRNAs	HCC Relevance
CYTOR (LINC00152)	miR-125a-5p	FAM169B, DRAM2,GCNT1,RFXANK, TRIM71, NLRC5, BAK1, SWSAP1, NPL, C19orf54	Oncogenesis, apoptosis resistance, metabolic reprogramming
	hsa-miR-107	RNF38, PPP6R2, TRIAP1, MED26, NPAS3, ANO3, ARIH2, NEK10, ARMC1, AGFG1	
	hsa-miR-33a-5p	ANO4, HMGA2, UBE2V2, CROT, CCNYL1, NPC1, RCAN1, SIRT6, YWHAH	
	hsa-miR-365a-3p	MEIS1, C5orf27, LPAR5, RP1-170O19.20, POC1B-GALNT4, ACVR1, HOXA9, USP48, TMEM183A, NFIB	
	hsa-let-7a-5p	HMGA2, ARID3B, LIN28B, FIGN, TRIM71,NR6A1, THRSP, USP44, FAM222B, IGDCC3	
UCA1	hsa-miR-136-3p	TRIAP1, RP11-1212A22.4, RPGR, SCP2D1, MEPE, COMMD2, RP11-422N16.3, FABP2, C19orf43, UGT2A3	FGFR1–ERK activation; drug resistance
	hsa-miR-212-5p	CNPY3, PRR14L, SPTBN2, HSCB, SRP9, SRRM4, PAFAH1B2, CACNA1E, XPO6, ACY1	
	hsa-miR-338-3p	FKBP1C, PVALB, DGKB, LGALSL, FKBP1A, UBE2Q1, CHTOP, NTPCR, C16orf87, ZBTB18	
	hsa-miR-216b-5p	C10orf11, CLGN, HELLS, NUDCD1, FAM151B, SAMD5, SLC25A35, DCUN1D4, YWHAB, CLK4	
	hsa-miR-326	KCNIP2, CEP85, CTRC, SYS1, ZNF394, HSD11B1, RGL3, DNAH10OS, CPLX2, KCNC1	
MALAT1	hsa-miR-122-5p	CTDNEP1, ALDOA, RFXAP, PLEKHB2, MASP1, CLIC4, SLC52A2,PRKRA, NICN1, AC002451.1	Inflammation, HIF-1α stabilisation, immune evasion
	hsa-miR-107	RNF38, PPP6R2, TRIAP1, MED26, NPAS3, ANO3, ARIH2, NEK10, ARMC1, AGFG1	
	hsa-miR-146a-5p	IGSF1, KBTBD4, PSMA4, CDKN2AIP, ZBTB2, IRAK1, SLC10A3, HNRNPD, ZDHHC13, NOVA1	
	hsa-miR-155-5p	ZNF385D, TMPRSS11BNL, VAV3, ETS1, ACTA1, ARID2, H3F3A, ZNF652, VMA21, ACTL7A	
	hsa-miR-181a-5p	ZNF780B, ZNF594, ZNF788, ZNF781, ZNF471, ZNF470, ZFP14, ZNF699, ZNF283, ZNF487	
SPRY4-IT1	hsa-miR-671-5p	C10orf25, SLC30A6, GDPD5,FAM83F, CCDC167, ZNF233, LGALS3BP, CPNE2, ST8SIA5, BCAP31	Fibrosis, epigenetic demethylation, splicing
	miR-101-5p	PTAR1, OGFRL1, ROR1, HSPE1, SNX10, SPIN2B, PBK, DMP1, NPY5R, OGN	
	hsa-miR-192-5p	FAM229B, EREG, LPAR4,NIPAL1, TYMS, MFAP3, GPR22, NKX2-5, H3F3B, TOR1AIP1	
	hsa-miR-29a-3p	COL1A1, TET3, TET1, MCMBP, PI15, TET2, ATAD2B, HRK, COL3A1,ELN	
	hsa-miR-143-3p	C1orf74, ITM2B, SLC25A15, ABHD14A, SLC30A8, MSI2, TMOD2, VAPB, TSC22D3, UBXN2B	
HULC	hsa-miR-2052	KCNJ6, XKR4,HOXA6, CYP4F3, STXBP5L, NECAB1,AMY2A, RGS17, EDNRB, C2ORF15	EMT, lipid metabolism, chemoresistance
	hsa-miR-200a-3p	ZNF385D, ZEB1, ZFR, ACOT7, TMEM170B, ZNHIT3, ZEB2, ANP32E, TADA1,RNF145	
	hsa-miR-107	RNF38, PPP6R2, TRIAP1, MED26,NPAS3, ANO3, ARIH2, NEK10, ARMC1,AGFG1	
	hsa-miR-372-5p	KIAA0101, PI15, ZFP69B, KRTAP13-3, NIM1, FUT9, SEC11C, LCMT2, SMIM9, FAR2	
	hsa-miR-9-3p	ZNF99, C10orf11, C12orf36, AC090186.1, ACTL6A, DSCC1, MZT1, NAA20, GTF2H5, RP11-542P2.1	
HOTAIR	hsa-let-7 g-5p	HMGA2, ARID3B, LIN28B, FIGN, TRIM71, NR6A1,THRSP, USP44, FAM222B, IGDCC3	Stemness, p53 regulation, epigenetic silencing
	hsa-miR-1-3p	CORO1C, SMIM14, ARPC3, PTPLAD1, TAGLN2, GJA1, ERMP1, SERP1, TMSB4X, MMD2	
	hsa-miR-122-5p	CTDNEP1, ALDOA, RFXAP, PLEKHB2, MASP1, CLIC4, SLC52A2, PRKRA, NICN1, AC002451.1	
	hsa-miR-125a-5p	FAM169B, DRAM2, GCNT1, RFXANK, TRIM71, NLRC5, BAK1, SWSAP1, NPL, C19orf54	
	hsa-miR-130a-3p	KDM2A, SLAIN1, MDM4, KLF7, PAN3, ENPP5, FBXO28, MYBL1, ACVR1, CPEB1	

Validated miRNA interaction data for uc001ncr and AF085935 are not currently available in DIANA-LncBase v3; these lncRNAs are therefore excluded from the supplementary network figures pending database updates and experimental confirmation

The expression of lncRNAs can be measured using qRT-PCR or RNA in situ hybridization. Their functions can be studied by silencing or overexpressing them through siRNA, shRNA, or CRISPR interference. Luciferase reporter assays confirm interactions between lncRNAs and miRNAs. RIP and ChIP techniques are used to study protein–lncRNA interactions. Western blotting helps detect changes in proteins involved in related signaling pathways. Computational tools are used to perform survival analysis (Kaplan–Meier), ROC curve analysis for biomarker accuracy, co-expression and ceRNA network construction, and pathway enrichment analysis, such as GSEA, to understand the biological functions of lncRNA targets [83].

New research approaches include single-cell RNA sequencing to analyze lncRNA expression at the cellular level and studying exosomal lncRNAs as potential non-invasive biomarkers in liquid biopsy samples. Therapeutic studies are also exploring antisense oligonucleotides and nanoparticle-based systems to target specific lncRNAs [84]. The main challenges in lncRNA research include achieving specific targeting, improving delivery methods, and translating experimental findings into clinical applications.

### Single-Cell RNA sequencing and spatial transcriptomics

Single-cell RNA (scRNA-seq) and spatial transcriptomics are revolutionizing the understanding of lncRNA biology in HCC by revealing expression heterogeneity that bulk sequencing methods mask. A landmark 2025 Nature Methods study used the TAR-scRNA-seq pipeline on data from 13 cancer types and identified 219,442 potential lncRNAs that showed cell-type-specific and spatially restricted expression patterns, confirming 94,795 previously unannotated lncRNA loci, including many candidates with microenvironment-specific expression in the liver cancer [85]. The work has important implications for the lncRNAs reviewed: expression profiles from bulk tumour tissue may mask the fact that individual lncRNAs are preferentially expressed in discrete cell subpopulations, such as tumour-associated macrophages, hepatic stellate cells, endothelial cells, or hepatic cancer stem cells, rather than bulk malignant hepatocytes.

For MALAT1 specifically, scRNA-seq studies of HCC have shown differential expression between different tumour cell clusters, suggesting that the prognostic associations of MALAT1 reported in bulk studies could be an aggregate of heterogeneous cell populations rather than an oncogenic mechanism shared across all tumour cells [86]. Likewise, HOTAIR-mediated epigenetic silencing could be spatially restricted to specific intratumoural zones, e.g., invasion fronts or hypoxic cores, rather than uniformly distributed. The HCCDB v2.0 database, comprising 5573 bulk transcriptomic samples from 26 HCC datasets and scRNA-seq data, provides a resource for cross-validation of bulk and single-cell lncRNA expression patterns in HCC [87].

Further studies of spatial transcriptomics revealed that the tumour microenvironment of HCC contains spatially separated immune and stromal subdomains, in which lncRNA regulatory networks might play a niche-specific role. scRNA-seq combined with spatial transcriptomics provides a powerful framework for identifying which cell populations within the HCC tumour are the primary drivers of lncRNA-mediated oncogenic effects, allowing more precise therapeutic targeting strategies [88].

### Exosomal and circulating lncRNAs as non-invasive biomarkers

Circulating long non-coding RNAs (lncRNAs) as free nucleic acids in plasma and serum or as exosomes derived from tumors are a promising class of minimally invasive biomarkers for HCC. Exosome-packaged lncRNAs are protected from RNase degradation by the lipid bilayer membrane and reflect the transcriptomic state of the parental tumour cells with substantial fidelity. The lncRNAs reviewed, HULC, HOTAIR, MALAT1, and SPRY4-IT1 have been detected in the serum of HCC patients with their expression levels significantly higher than those of hepatic cirrhosis, chronic hepatitis, and healthy control groups [89]. Of particular note, AF085935 was specifically elevated in the serum of patients with HBV- and HCV-positive HCC and may be useful as a liquid biopsy-specific biomarker for viral etiology [90].

However, there are some critical and often underappreciated challenges in the clinical translation of circulating lncRNA biomarkers. First, Standardisation of exosome isolation methods, including ultracentrifugation, precipitation-based kits, and size-exclusion chromatography, yields markedly different lncRNA yield and purity profiles, preventing meaningful cross-study comparisons. Second, suitable reference genes for normalization of circulating lncRNA quantification have not been standardised [91]. Commonly used reference RNAs (miR-16, RNU6B, GAPDH) show variable stability in plasma/serum and may introduce systematic bias. Third, most published studies involve relatively small cohorts (*n* < 100), which limits statistical power and generalizability. Fourth, comparative sensitivity/specificity data relative to AFP, the current standard clinical biomarker, are available for very few of the lncRNAs we reviewed, making it difficult to assess their incremental diagnostic value [92]. Table 5 provides a structured comparison of the diagnostic and prognostic performance of the reviewed lncRNAs based on available clinical data.

**Table 5:** Comparative diagnostic and prognostic performance of key lncRNAs in HCC

lncRNA	Sample Type	AUC (single marker)	Sensitivity (%)	Specificity (%)	AUC (panel + AFP)	Panel composition	Prognostic association	Ref
HOTAIR	Serum	**0.991**	96. 7	95.0	**0.998**	HOTAIR + BRM + ICR + AFP	Independent predictor of post-transplant recurrence (HR 3.564, *p* = 0.001); correlated with TNM/BCLC stage, vascular invasion, PVTT, tumour size	[78, 93]
MALAT1	Serum / Plasma	0.79 (vs. HC); 0.70 (vs. cirrhosis)	Meta: 69%	Meta: 85%	0.968	MALAT1 + HOTTIP + AFP	Associated with multiple tumour lesions, HCV infection, elevated AST/AFP; shorter overall survival (OS)	[44, 94]
HULC	Plasma / Tissue	0.86 (tissue vs. HC); 0.78 (serum vs. HC)	NR (tissue study)	NR (tissue study)	0.87 (HULC + CYTOR); 0.89 (+ AFP)	HULC + Linc00152 + AFP	Higher expression in HBV+ patients and advanced Edmondson grade correlated with OS; liver-specific expression reduces background noise	[89, 95]
UCA1	Serum	0.91 (vs. cirrhosis)	85.0	83.3	0.912	Linc00152 (CYTOR) + UCA1 + AFP	Correlated with vascular invasion, late TNM stage; high UCA1 linked to poor OS, tumour size and metastasis (meta-analysis of 7 studies); independent prognostic indicator (serum LINC00152 HR = 2.23)	[89, 96]
CYTOR (LINC00152)	Serum / Tissue	0.877 (serum, single marker, best in panel study)	81.7	63.3	0.906 (Linc00152 + AFP)	Linc00152 + AFP	High expression correlated with advanced TCGA stage (*p* = 8.07 × 10⁻⁷), T-classification (*p* = 4.73 × 10⁻⁷), metastasis; worse OS on Kaplan–Meier (TCGA cohort); independent indicator of poor OS in serum (HR = 2.23, *p* = 0.03)	[61, 89, 96]
SPRY4-IT1	Serum	Significantly elevated (specific AUC NR in single-marker studies)	NR	NR	NR (included in multi-lncRNA panel)	Part of 8-lncRNA serum panel (Huang et al.)	Elevated serum levels correlate with ALT; poor prognosis and metastasis in tissue studies; EZH2-mediated H3K27me3 silencing of E-cadherin; Twist1 upregulation	[97]
uc001ncr (TUC338)	Serum	0.8859 (serum, training cohort)	NR	NR	0.9494 / 0.9491 (training / validation)	uc001ncr + AX800134 panel (HBV-positive HCC)	Advanced tumour invasion and metastasis; HBV-specific upregulation; ≤AFP 400 ng/mL AUC 0.9371 (training) / 0.9527 (validation); early HCC (BCLC 0 + A) AUC 0.9450/0.9564	[98]
AF085935 (LINC01152)	Serum	0.96 (HCC vs. healthy controls)	NR	NR	0.86 (HCC vs. HBV patients)	AF085935 alone; also studied with uc003wbd	Upregulated specifically in HBV/HCV-positive HCC; discriminates HCC from HBV carriers (AUC 0.86) and from healthy controls (AUC 0.96); differential expression reflects viral burden	[99]

*AUC *area under the receiver operating characteristic curve, *HC *healthy controls, *HBV *hepatitis B virus, *HCV *hepatitis C virus, *HR *hazard ratio, *NR *not reported, *OS *overall survival, *PVTT *portal vein tumour thrombus, *Sn *sensitivity, *Sp *specificity, *TCGA *The Cancer Genome Atlas, *TNM *tumour–node–metastasis staging

## Therapeutic strategies and clinical translation challenges

### RNA interference and antisense oligonucleotides

RNA interference (RNAi) approaches, including small interfering RNAs (siRNAs), short hairpin RNA (shRNA), and nucleoside-based antisense oligonucleotides (ASOs), represent the most straightforward tools to directly alter lncRNA expression for therapeutic purposes. siRNA and shRNA approaches provide robust target knockdown but have poor stability in biological fluids, rapid clearance, and inefficient cellular uptake when used without a carrier [100]. LNA-based ASOs, particularly LNA gapmeR ASOs, have much better pharmacokinetic profiles and have been used experimentally to target MALAT1 in a sequence-specific manner in preclinical cancer models, achieving significant inhibition of cancer growth [101]. However, a major off-target issue relating to all RNAi-based strategies arises because one single siRNA sequence can target hundreds of partly complementary transcripts throughout the transcriptome, raising hepatotoxicity concerns when given to HCC patients with pre-existing cirrhosis or viral hepatitis. Consequently, conducting a thorough transcriptome-wide off-target profiling is an important requirement for any lncRNA-targeting therapeutic candidate that is moving to clinical evaluation [100].

### CRISPR-based approaches

Precise modulation of lncRNA without permanent genome editing is possible with CRISPR-based technologies. By directing guide RNAs to promoter regions, CRISPRi (interference), which uses a catalytically dead dCas9 linked to transcriptional repressors (KRAB domain), allows for the reversible and powerful transcriptional suppression of lncRNA genes. On the other hand, by attracting transcriptional activators, CRISPRa (activation) can upregulate tumor-suppressive lncRNAs. These methods are especially useful for big lncRNAs, like MALAT1 (~ 8 kb) and HOTAIR (~ 2.1 kb), whose transcript size prevents them from being packaged into traditional viral vectors [102]. Delivery vehicle limitations continue to be a significant obstacle: full-length MALAT1 cannot be delivered using adeno-associated virus (AAV) vectors due to their packaging limit of roughly 4.7 kb. Non-viral lipid nanoparticle (LNP) or polymer-based delivery of CRISPR machinery represents a clinically relevant alternative for hepatic targets, given the natural tropism of intravenously delivered LNPs for the liver [103].

### Nanoparticle-based delivery platforms

Due to their effective application in the administration of mRNA vaccines, lipid nanoparticles (LNPs) have become the most popular clinically validated delivery method for nucleic acid treatments. LNP-mediated delivery provides several benefits for lncRNA-targeted therapy in HCC: pH-responsive ionisable lipid formulations facilitate endosomal escape, improving intracellular nucleic acid delivery; GalNAc (N-acetylgalactosamine) conjugation to the nanoparticle surface allows active targeting of hepatocytes via asialoglycoprotein receptors; and hepatic first-pass metabolism paradoxically promotes liver-selective drug accumulation [104]. A 2024 study showed that a cisplatin prodrug and an endosomal pH-responsive nanoparticle co-delivering siRNA against lncMALAT1 produced a synergistic reversal of cisplatin resistance in HCC cells both in vitro and in vivo [105]. In 2024, ionisable LNP systems that encapsulated ASOs against HOTAIR and MALAT1 showed successful silencing in a variety of cancer cell lines [106]. Despite these encouraging preclinical results, no lncRNA-targeting nanoparticle therapy has progressed beyond Phase I evaluation for any solid tumour indication, and none specifically for HCC—a reflection of the formidable translational barriers outlined below.

### Clinical feasibility and the translational gap

Despite growing and mechanistically compelling preclinical evidence, no lncRNA-targeting medication has progressed to Phase III clinical trials for HCC. This translational gap is a result of several interrelated obstacles. First, the predictive accuracy of murine HCC models is limited by the low interspecies sequence conservation of most lncRNAs, such as CYTOR, uc001ncr, and AF085935. This raises questions regarding whether preclinical efficacy translates to human disease. Second, because lncRNAs like CYTOR have multiple isoforms, it becomes unclear whether a particular isoform should be targeted because distinct isoforms may have competing or diverging functions [107]. Third, cirrhosis, portal hypertension, or active viral hepatitis are common conditions that impede hepatic clearance of nucleic acid constructs and increase susceptibility to inflammatory responses triggered by double-stranded RNA intermediates. As a result, the risk of hepatotoxicity is significantly increased in the HCC patient population. Fourth, therapeutic siRNA or ASO constructs that stimulate the immune system through innate RNA-sensing pathways (RIG-I, TLR3, TLR7) pose a risk of systemic toxicity that must be reduced by chemical modification (phosphorothioate linkages, 2’-O-methyl modifications). Fifth, no lncRNA treatment program has standardized patient categorization based on baseline lncRNA expression before enrollment, which limits the capacity to select individuals most likely to benefit. Successful clinical translation will require integrated toxicogenomic profiling, appropriate patient stratification by lncRNA expression and HCC etiology, and rational combination strategies with existing standard-of-care agents such as sorafenib, lenvatinib, or atezolizumab/bevacizumab [108].

## Future perspectives and knowledge gaps

The majority of reported HCC-associated lncRNAs, including several reviewed here, are primarily supported by single-laboratory studies in small patient cohorts, often using cell line overexpression systems rather than genetically defined in vivo models. In contrast, a small number of well-validated molecules (HULC, MALAT1, HOTAIR) have accumulated robust multi-cohort evidence. To validate expression-outcome associations, establish clinically actionable cut-off values, and evaluate lncRNA performance across various HCC etiologies (HBV, HCV, NAFLD, alcohol), large, multicenter cohort studies with prospective sample collection are urgently needed. This is especially true in high-burden regions like South Asia, East Africa, and the Middle East, where HBV-associated HCC is common and there is a lack of biomarker data. To enable cross-laboratory comparison, pre-analytical protocols for circulating lncRNA quantification must be standardized. This includes reaching an agreement on the selection of reference genes for normalization, exosome isolation techniques, and minimum reporting standards similar to the MIQE guidelines for qPCR. Beyond what is possible with lncRNA alone, the diagnostic and prognostic efficacy of multivariate prediction models may be significantly increased by integrating lncRNA expression profiles with current clinical data (AFP levels, imaging, viral load, and BCLC stage).

An unexplored area with direct clinical relevance for individualized HCC management is the potential of exosomal and circulating lncRNAs as real-time treatment monitoring biomarkers, reflecting dynamic changes in tumor transcriptome in response to sorafenib, immune checkpoint inhibition, or loco-regional therapy. Finally, the systematic application of scRNA-seq and spatial transcriptomics to characterize lncRNA expression at cell-type and spatially resolved levels within HCC tumour and peri-tumoral tissue will be essential for identifying which cellular compartments most meaningfully contribute to lncRNA-driven oncogenesis and which represent the most productive therapeutic targets.

## Conclusion

In the larger framework of HCC pathogenesis, this review provides a targeted and mechanistically based examination of eight lncRNAs: CYTOR, UCA1, MALAT1, SPRY4-IT1, uc001ncr, AF085935, HULC, and HOTAIR. Together, these molecules control key carcinogenic pathways, including PI3K/AKT/mTOR, Wnt/β-catenin, NF-κB, and EMT. They also show roles as therapeutic targets and diagnostic and prognostic biomarkers that are different from and possibly complementary to current clinical markers.

This synthesis leads to a number of important conclusions. First, while emerging candidates (uc001ncr, AF085935) need much larger prospective validation studies, well-validated lncRNAs (HULC, HOTAIR, MALAT1) show repeatable clinical associations across independent cohorts, including serum-based AUC values of 0.79–0.998, establishing a solid foundation for biomarker development. Second, the majority of the researched lncRNAs include ceRNA-based processes, but the stoichiometric validity of ceRNA interactions at endogenous expression levels has not yet been thoroughly investigated. This caveat must be addressed before ceRNA-based models are used in clinical settings. Third, delivery obstacles, hepatotoxicity risks in the cirrhotic HCC population, off-target silencing, and the lack of Phase III clinical evidence limit the therapeutic translation of lncRNA biology. Fourth, lncRNA expression profiles are influenced by viral etiology (HBV or HCV), especially for uc001ncr and AF085935. This suggests that etiology-stratified biomarker research is crucial in high-burden endemic areas. Fifth, cell-type-specific and spatially limited regulatory activities that are hidden by bulk expression analyses are starting to be revealed by the integration of single-cell RNA sequencing and spatial transcriptomics into lncRNA research, offering more accurate mechanistic understanding and more logical therapeutic targeting. There is still much to learn about the lncRNA landscape in HCC. Only a small portion of the regulatory complexity behind liver cancer is represented by the eight molecules discussed here. Large multicenter validation cohorts, standardized liquid biopsy procedures, integration with multi-omics data, and clinical trials using lncRNA expression-based patient classification are all necessary for future advancements. With these developments, lncRNA-based biomarkers and treatment approaches have the potential to significantly improve hepatocellular carcinoma diagnosis and treatment.

## Supplementary Information

Supplementary Material 1: Supplementary Figures S1–S6. predicted lncRNA–miRNA–mRNA ceRNA interaction networks for CYTOR (S1), UCA1 (S2), MALAT1 (S3), SPRY4-IT1 (S4), HULC (S5), and HOTAIR (S6) in hepatocellular carcinoma. Each panel displays the full list of predicted target mRNAs for each miRNA axis as retrieved from DIANA-LncBase v3 (lncRNA–miRNA interactions, accessed January 2026) and TargetScan v8.0 (miRNA–mRNA target predictions, accessed January 2026). Solid arrows indicate lncRNA–miRNA sponging (ceRNA mechanism). Dashed arrows indicate miRNA-mediated mRNA silencing that is de-repressed upon lncRNA sponging. All interactions are computationally predicted; experimental validation in HCC-specific models is required to confirm functional significance. Note on uc001ncr and AF085935: validated miRNA interaction data are not yet available in current databases for these two lncRNAs.

## Data Availability

No datasets were generated or analysed during the current study.
