# Supplementary figures and images for: Decoding the functional landscape of long non-coding RNAs in hepatocellular carcinoma: molecular mechanisms, clinical implications, and therapeutic prospects
Source: J Egypt Natl Canc Inst. 2026 Jun 29;38:39. doi: 10.1186/s43046-026-00380-9 (PMC13315050; doi:10.1186/s43046-026-00380-9)

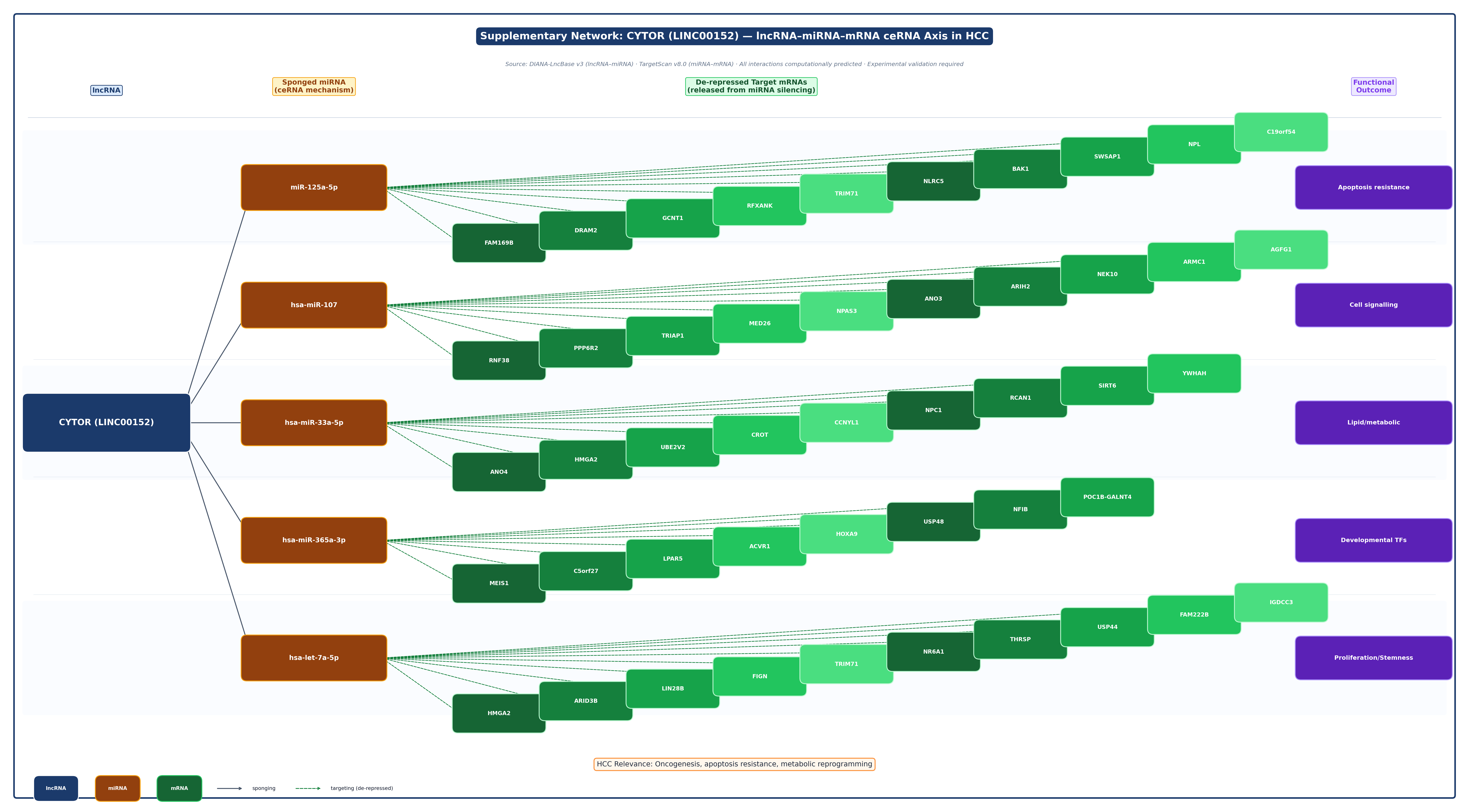

Supplement: Supplementary file 1 — Supplementary Material 1: Supplementary Figures S1–S6. predicted lncRNA–miRNA–mRNA ceRNA interaction networks for CYTOR (S1), UCA1 (S2), MALAT1 (S3), SPRY4-IT1 (S4), HULC (S5), and HOTAIR (S6) in hepatocellular carcinoma. Each panel displays the full list of predicted target mRNAs for each miRNA axis as retrieved from DIANA-LncBase v3 (lncRNA–miRNA interactions, accessed January 2026) and TargetScan v8.0 (miRNA–mRNA target predictions, accessed January 2026). Solid arrows indicate lncRNA–miRNA sponging (ceRNA mechanism). Dashed arrows indicate miRNA-mediated mRNA silencing that is de-repressed upon lncRNA sponging. All interactions are computationally predicted; experimental validation in HCC-specific models is required to confirm functional significance. Note on uc001ncr and AF085935: validated miRNA interaction data are not yet available in current databases for these two lncRNAs. [file 43046_2026_380_MOESM1_ESM.zip › Supplementary/Suppl_Network_CYTOR_LINC00152.png]

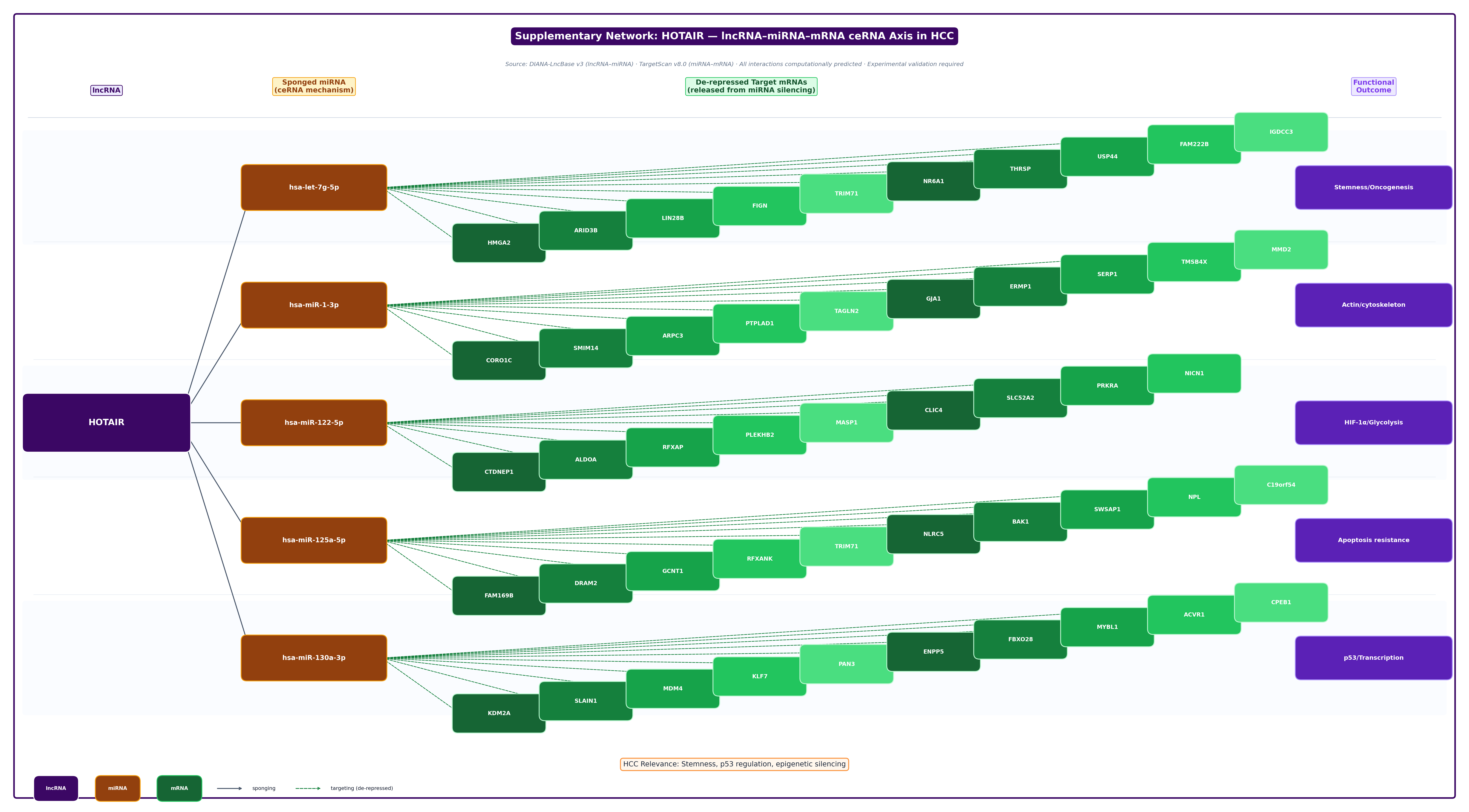

Supplement: Supplementary file 1 — Supplementary Material 1: Supplementary Figures S1–S6. predicted lncRNA–miRNA–mRNA ceRNA interaction networks for CYTOR (S1), UCA1 (S2), MALAT1 (S3), SPRY4-IT1 (S4), HULC (S5), and HOTAIR (S6) in hepatocellular carcinoma. Each panel displays the full list of predicted target mRNAs for each miRNA axis as retrieved from DIANA-LncBase v3 (lncRNA–miRNA interactions, accessed January 2026) and TargetScan v8.0 (miRNA–mRNA target predictions, accessed January 2026). Solid arrows indicate lncRNA–miRNA sponging (ceRNA mechanism). Dashed arrows indicate miRNA-mediated mRNA silencing that is de-repressed upon lncRNA sponging. All interactions are computationally predicted; experimental validation in HCC-specific models is required to confirm functional significance. Note on uc001ncr and AF085935: validated miRNA interaction data are not yet available in current databases for these two lncRNAs. [file 43046_2026_380_MOESM1_ESM.zip › Supplementary/Suppl_Network_HOTAIR.png]

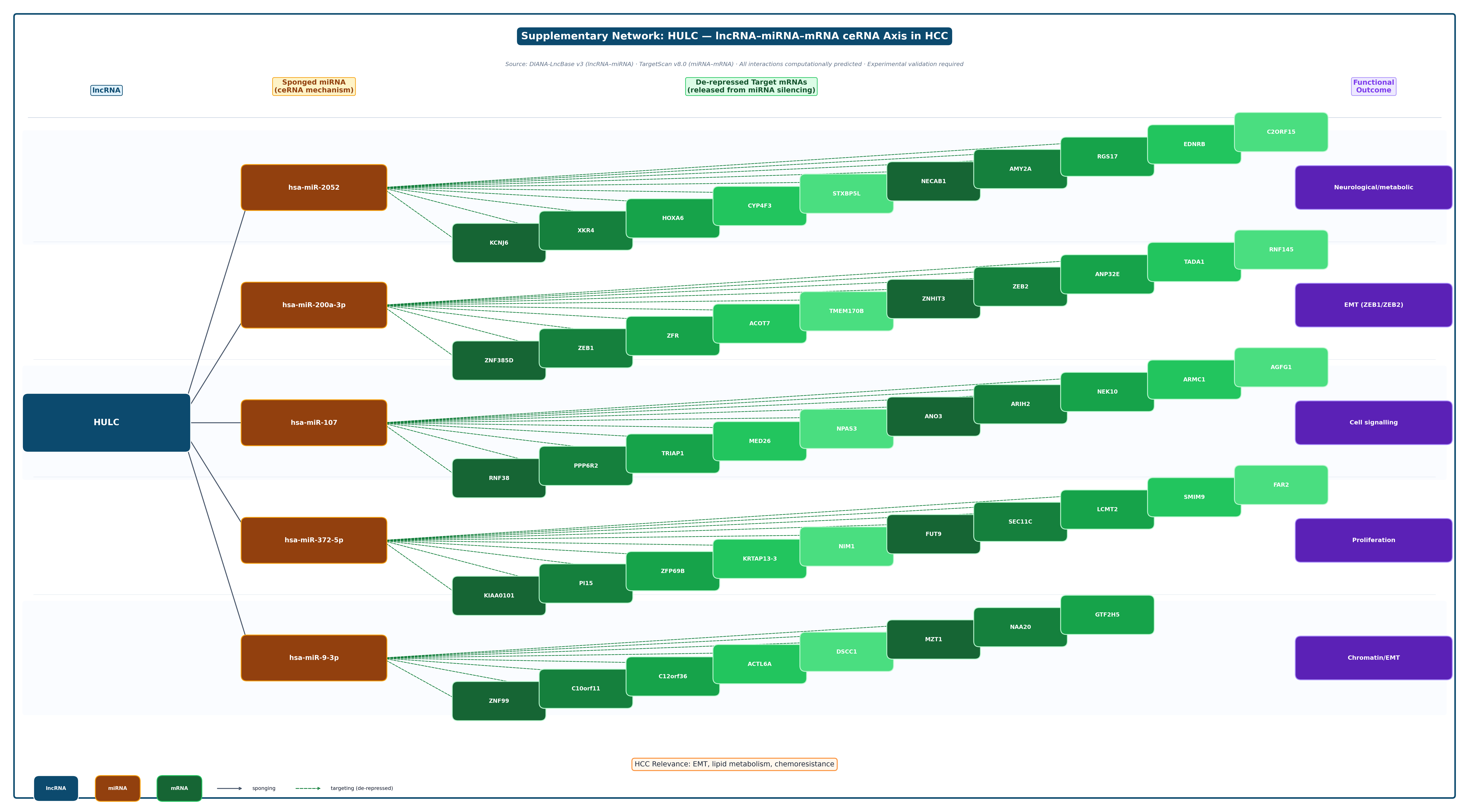

Supplement: Supplementary file 1 — Supplementary Material 1: Supplementary Figures S1–S6. predicted lncRNA–miRNA–mRNA ceRNA interaction networks for CYTOR (S1), UCA1 (S2), MALAT1 (S3), SPRY4-IT1 (S4), HULC (S5), and HOTAIR (S6) in hepatocellular carcinoma. Each panel displays the full list of predicted target mRNAs for each miRNA axis as retrieved from DIANA-LncBase v3 (lncRNA–miRNA interactions, accessed January 2026) and TargetScan v8.0 (miRNA–mRNA target predictions, accessed January 2026). Solid arrows indicate lncRNA–miRNA sponging (ceRNA mechanism). Dashed arrows indicate miRNA-mediated mRNA silencing that is de-repressed upon lncRNA sponging. All interactions are computationally predicted; experimental validation in HCC-specific models is required to confirm functional significance. Note on uc001ncr and AF085935: validated miRNA interaction data are not yet available in current databases for these two lncRNAs. [file 43046_2026_380_MOESM1_ESM.zip › Supplementary/Suppl_Network_HULC.png]

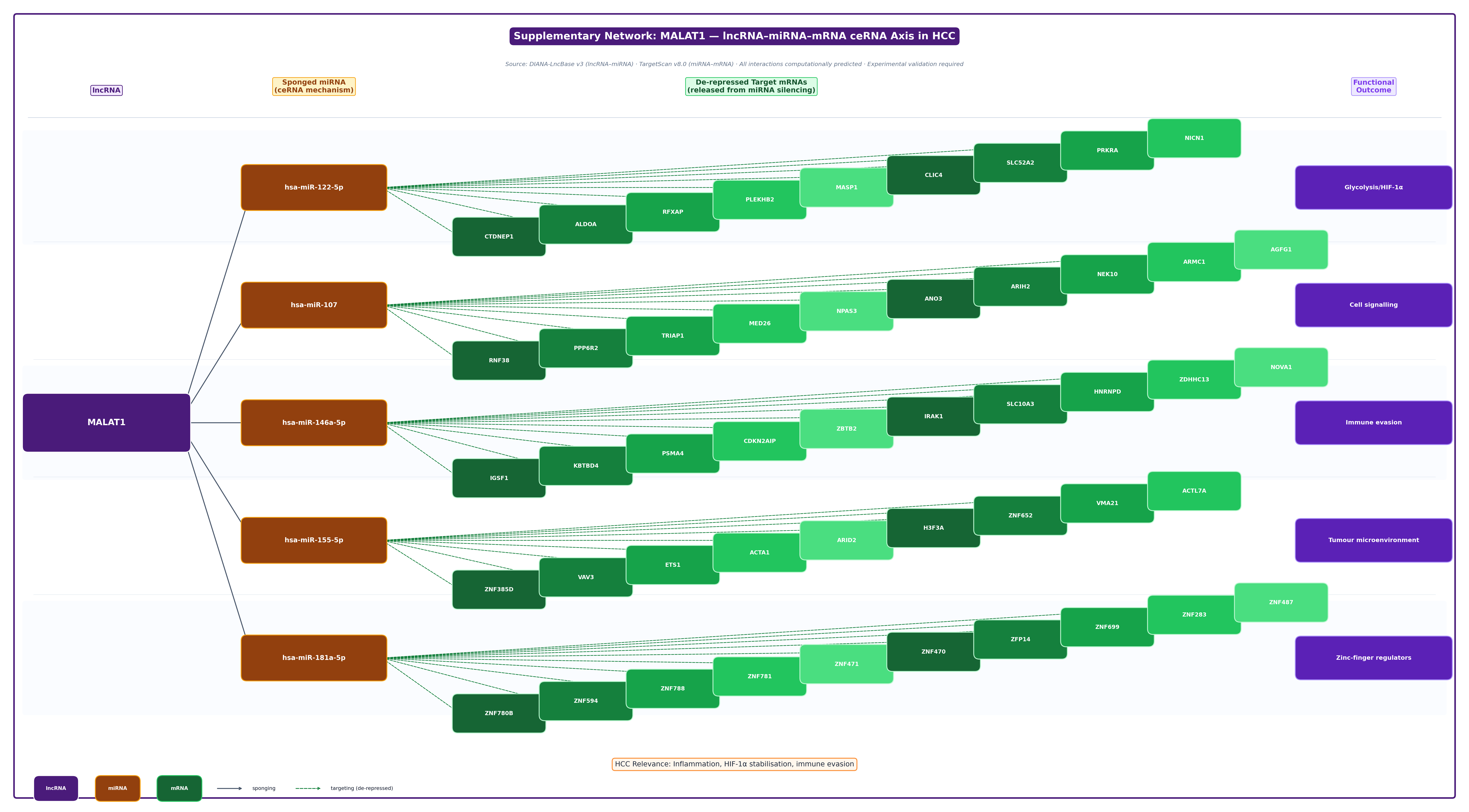

Supplement: Supplementary file 1 — Supplementary Material 1: Supplementary Figures S1–S6. predicted lncRNA–miRNA–mRNA ceRNA interaction networks for CYTOR (S1), UCA1 (S2), MALAT1 (S3), SPRY4-IT1 (S4), HULC (S5), and HOTAIR (S6) in hepatocellular carcinoma. Each panel displays the full list of predicted target mRNAs for each miRNA axis as retrieved from DIANA-LncBase v3 (lncRNA–miRNA interactions, accessed January 2026) and TargetScan v8.0 (miRNA–mRNA target predictions, accessed January 2026). Solid arrows indicate lncRNA–miRNA sponging (ceRNA mechanism). Dashed arrows indicate miRNA-mediated mRNA silencing that is de-repressed upon lncRNA sponging. All interactions are computationally predicted; experimental validation in HCC-specific models is required to confirm functional significance. Note on uc001ncr and AF085935: validated miRNA interaction data are not yet available in current databases for these two lncRNAs. [file 43046_2026_380_MOESM1_ESM.zip › Supplementary/Suppl_Network_MALAT1.png]

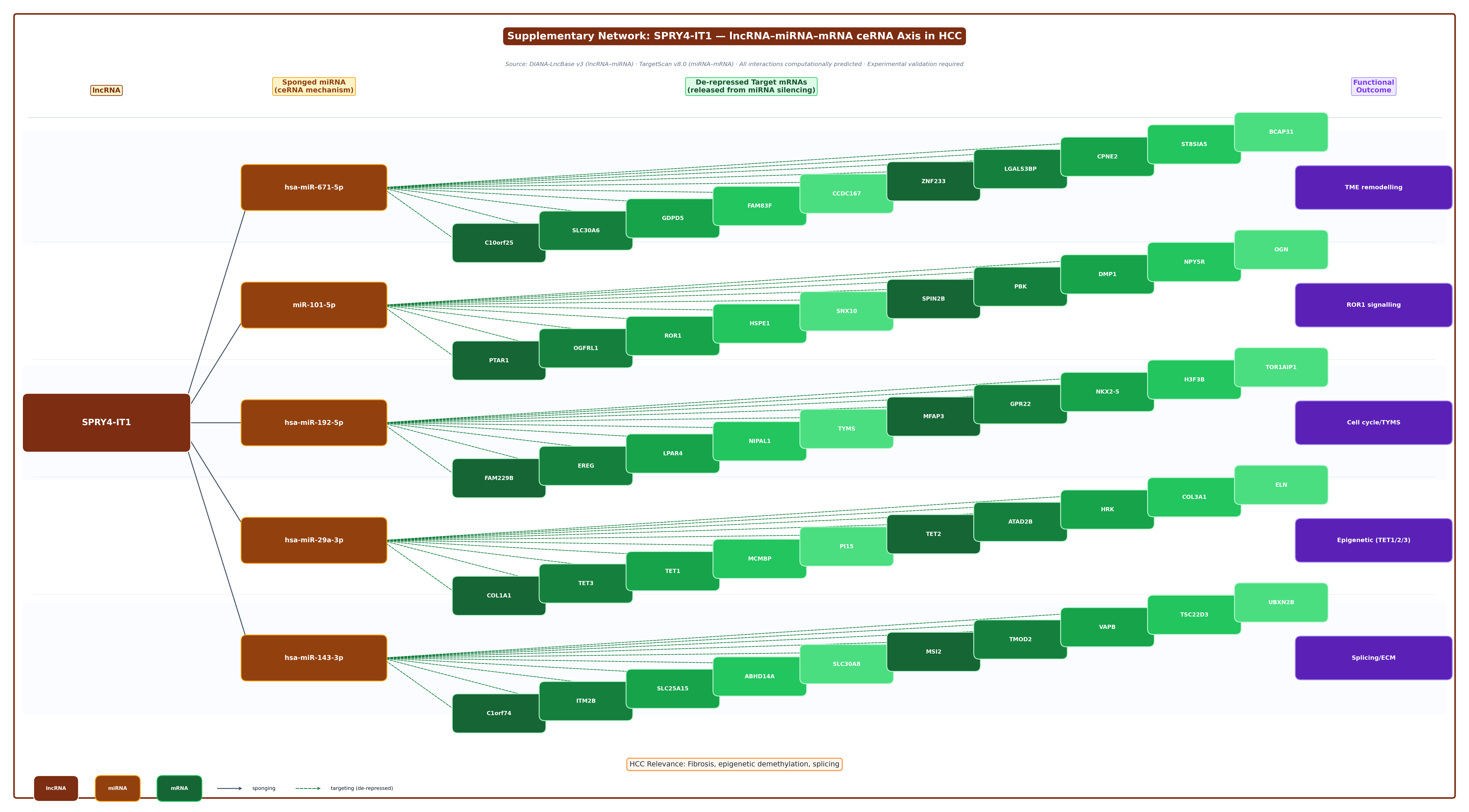

Supplement: Supplementary file 1 — Supplementary Material 1: Supplementary Figures S1–S6. predicted lncRNA–miRNA–mRNA ceRNA interaction networks for CYTOR (S1), UCA1 (S2), MALAT1 (S3), SPRY4-IT1 (S4), HULC (S5), and HOTAIR (S6) in hepatocellular carcinoma. Each panel displays the full list of predicted target mRNAs for each miRNA axis as retrieved from DIANA-LncBase v3 (lncRNA–miRNA interactions, accessed January 2026) and TargetScan v8.0 (miRNA–mRNA target predictions, accessed January 2026). Solid arrows indicate lncRNA–miRNA sponging (ceRNA mechanism). Dashed arrows indicate miRNA-mediated mRNA silencing that is de-repressed upon lncRNA sponging. All interactions are computationally predicted; experimental validation in HCC-specific models is required to confirm functional significance. Note on uc001ncr and AF085935: validated miRNA interaction data are not yet available in current databases for these two lncRNAs. [file 43046_2026_380_MOESM1_ESM.zip › Supplementary/Suppl_Network_SPRY4-IT1.png]

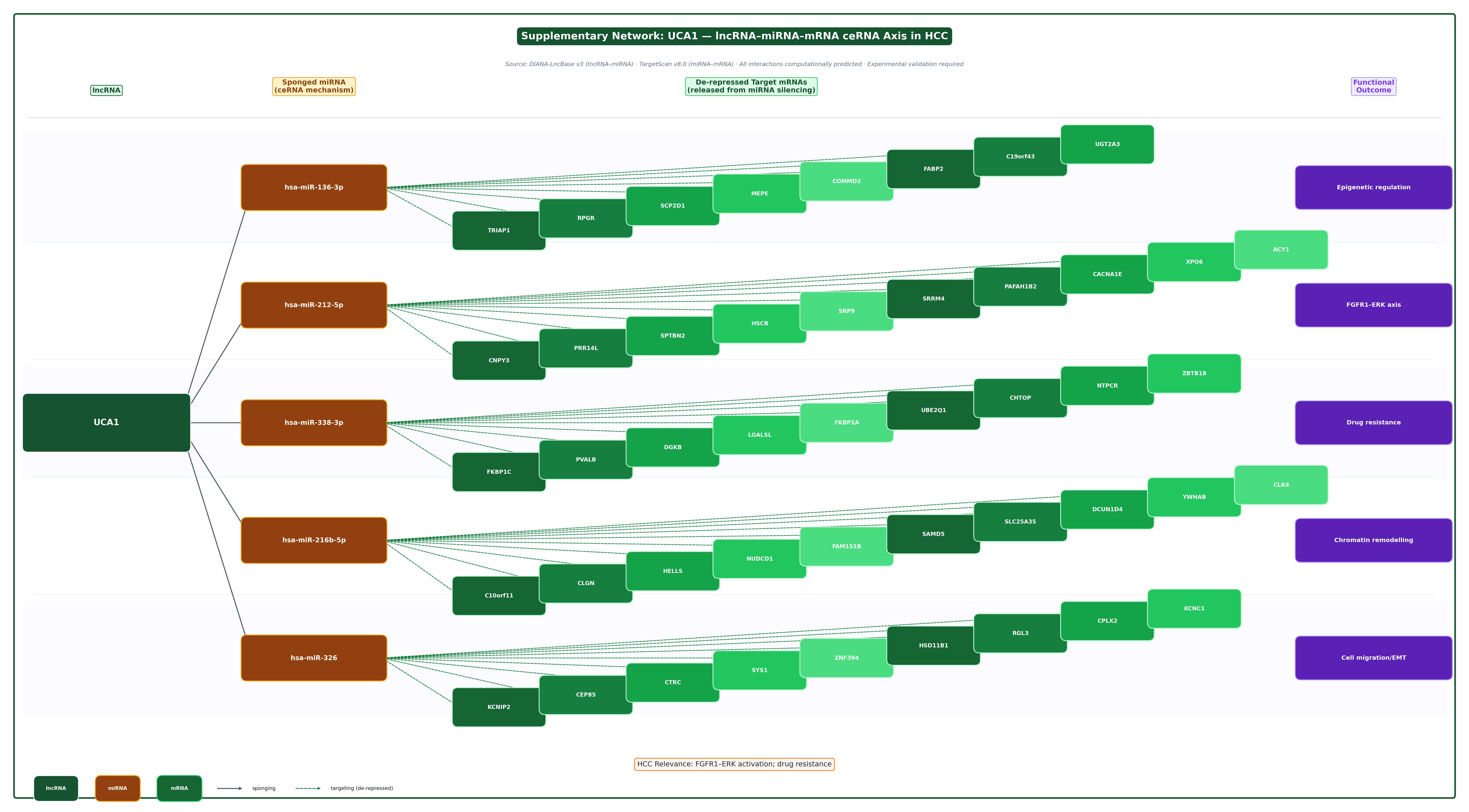

Supplement: Supplementary file 1 — Supplementary Material 1: Supplementary Figures S1–S6. predicted lncRNA–miRNA–mRNA ceRNA interaction networks for CYTOR (S1), UCA1 (S2), MALAT1 (S3), SPRY4-IT1 (S4), HULC (S5), and HOTAIR (S6) in hepatocellular carcinoma. Each panel displays the full list of predicted target mRNAs for each miRNA axis as retrieved from DIANA-LncBase v3 (lncRNA–miRNA interactions, accessed January 2026) and TargetScan v8.0 (miRNA–mRNA target predictions, accessed January 2026). Solid arrows indicate lncRNA–miRNA sponging (ceRNA mechanism). Dashed arrows indicate miRNA-mediated mRNA silencing that is de-repressed upon lncRNA sponging. All interactions are computationally predicted; experimental validation in HCC-specific models is required to confirm functional significance. Note on uc001ncr and AF085935: validated miRNA interaction data are not yet available in current databases for these two lncRNAs. [file 43046_2026_380_MOESM1_ESM.zip › Supplementary/Suppl_Network_UCA1.png]
